# Crystal structure of racemic chloramphenicol

**DOI:** 10.1107/S2056989026008765

**Published:** 2026-08-28

**Authors:** Odil Choriev, Jamshid Ashurov, Batirbay Torambetov, Tuncer Hökelek, Alebel N. Belay, Khudayar I. Hasanov, Reyhan Sh. Hajiyeva

**Affiliations:** a Institute of Bioorganic Chemistry, Academy of Sciences of Uzbekistan, Mirzo Ulugbek Str. 83, Tashkent 100125, Uzbekistan; b National University of Uzbekistan named after Mirzo Ulugbek 4 University St Tashkent 100174 Uzbekistan; c Hacettepe University, Department of Physics, 06800 Beytepe-Ankara, Türkiye; d Department of Chemistry, Bahir Dar University, PO Box 79, Bahir Dar, Ethiopia; e Azerbaijan Medical University, Scientific Research Centre (SRC), A. Kasumzade St. 14, AZ 1022, Baku, Azerbaijan; f Department of Technology of Chemical and Inorganic Substances, Azerbaijan State Oil and Industry University, Azadliq Avenue 34, AZ1010, Baku, Azerbaijan; University of Aberdeen, United Kingdom

**Keywords:** chloramphenicol, crystal structure, noncovalent inter­actions

## Abstract

The racemic title compound {systematic name: 2,2-di­chloro-*N*-[(1*R*,2*R*)-1,3-dihy­droxy-1-(4-nitro­phen­yl)propan-2-yl]acetamide}, C_11_H_12_Cl_2_N_2_O_5_, crystallizes in the space group *P*1. In the crystal, O—H⋯O, N—H⋯O and C—H⋯O hydrogen bonds link the mol­ecules into a three-dimensional architecture, enclosing *R*^2^_2_(14), *R*^2^_2_(12), *R*^2^_2_(10) and *R*^4^_4_(4) loops. The title compound complements the known ortho­rhom­bic form of natural (homochiral) chloramphenicol [Acharya *et al.* (1979 ▸). Acta Cryst. B**35**, 1360–1363], which crystallizes in space group *C*222_1_. The Hirshfeld surface analysis of the crystal structure indicates that the most important contributions for the crystal packing are from H⋯O/O⋯H (39.4%), H⋯H (21.7%), H⋯C/C⋯H (15.5%) and Cl⋯C/C⋯Cl (8.3%) inter­actions.

## Abstract 2

The racemic title compound crystallizes in space group *P*1 compared to *C*222_1_ for the chiral natural form.

## 1. Chemical context

Chloramphenicol, C_11_H_12_Cl_2_N_2_O_5_, was originally isolated from the bacteria *Streptomyces venezuelae* in 1948 and has been used to treat bacterial conjunctivitis, which is a bacterial infection involving the mucous membrane of the surface of the eye (Ehrlich *et al.*, 1947 ▸; Feder *et al.*, 1981 ▸). The crystal structure of natural chloramphenicol has been reported several times in the ortho­rhom­bic space group *C*222_1_ (Acharya *et al.*, 1979 ▸; Chatterjee *et al.*, 1979 ▸; Dunitz, 1952 ▸; Staples, 2022 ▸; Sundaralingam *et al.*, 1971 ▸). In this work, we report the crystal structure of synthetic (racemic) chloramphenicol (**I**), and compare it with the natural form.
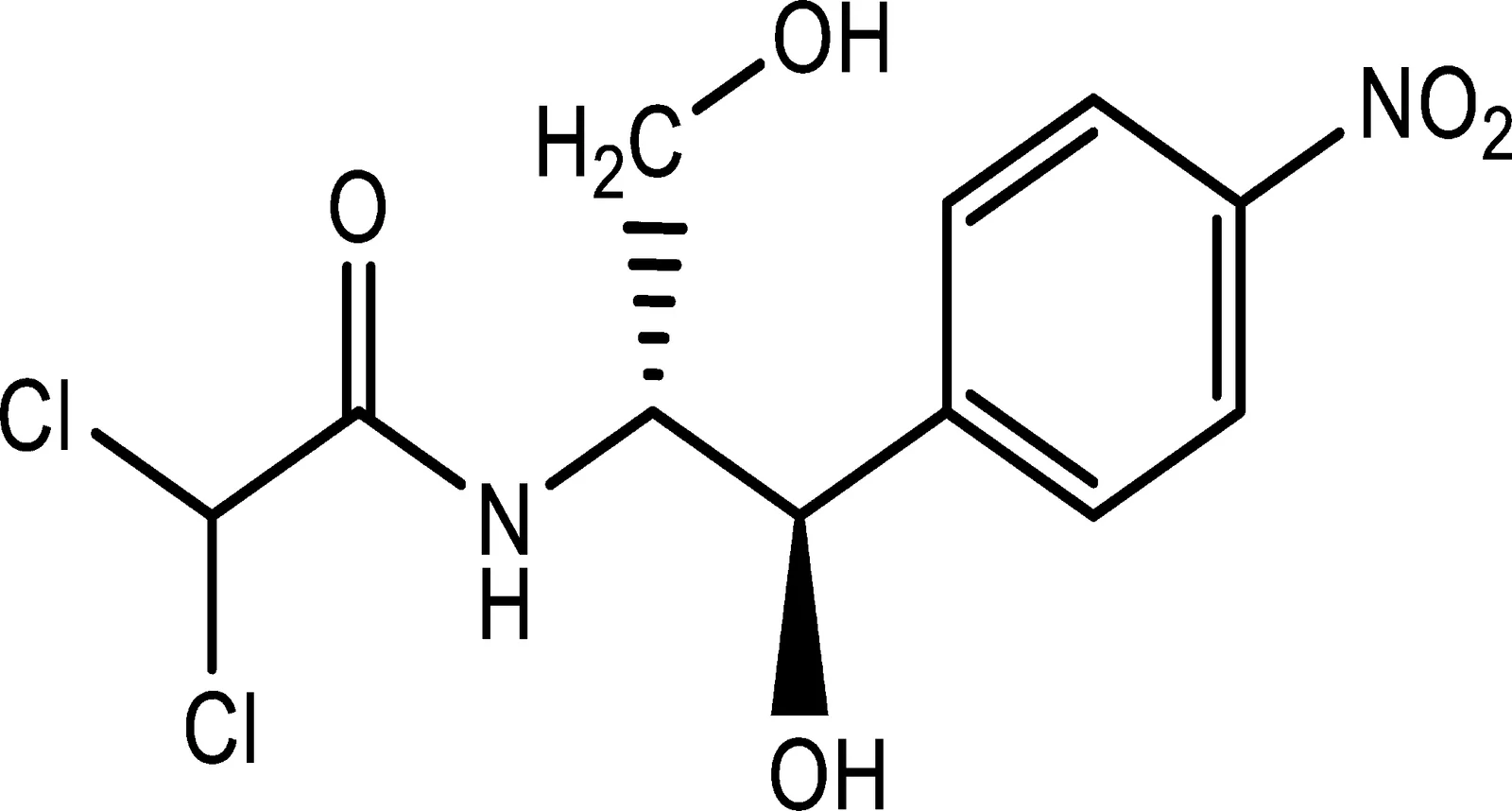


## 2. Structural commentary

**Figure 1:**
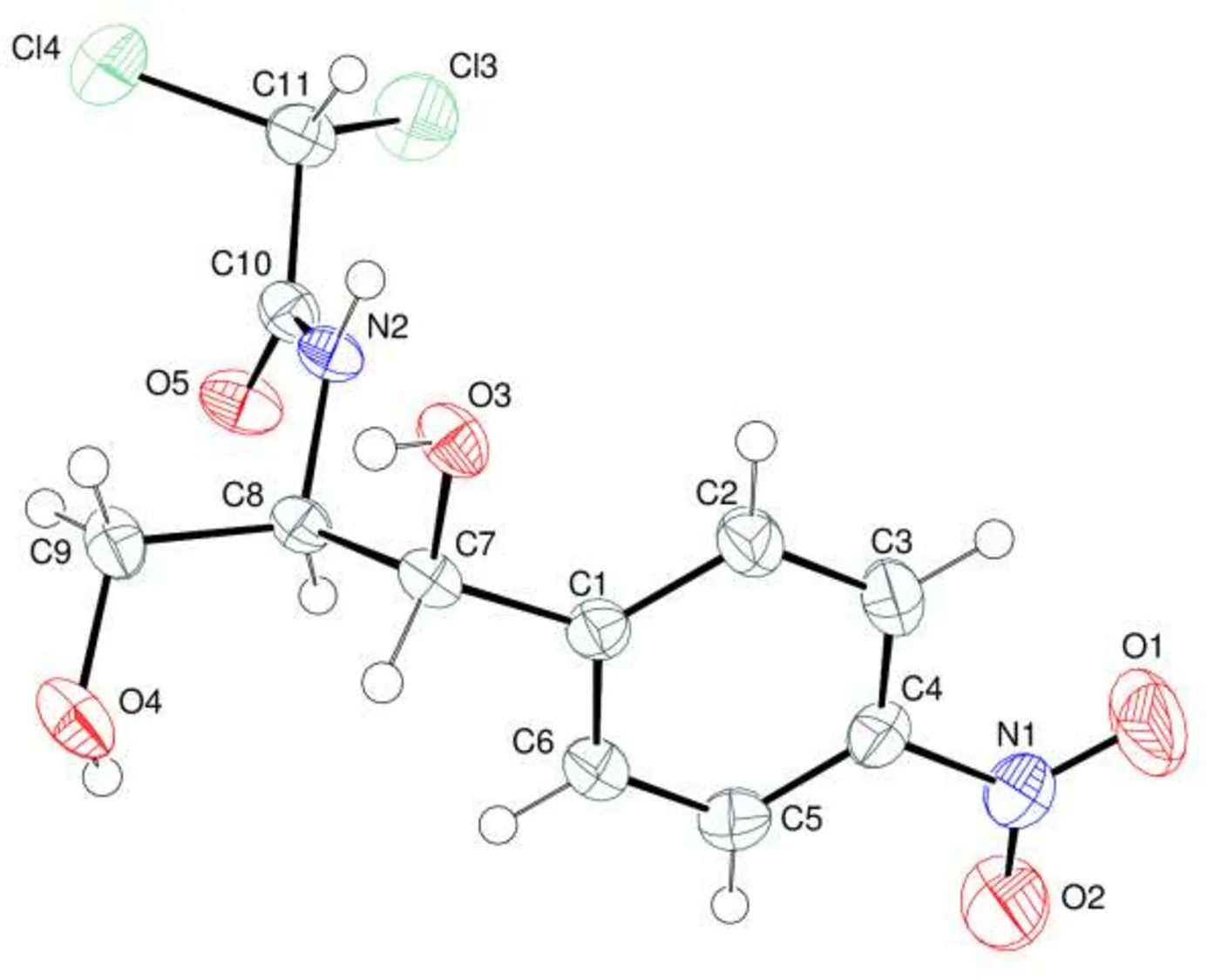
The mol­ecular structure of (**I**) showing 50% probability ellipsoids.

Compound (**I**) crystallizes in the centrosymmetric triclinic space group *P*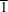 with one mol­ecule in the asymmetric unit. The mol­ecule consists of *p*-nitro­benzene, dichloracetyl and 2-amino-propane­diol fragments (Fig. 1 ▸), where the dichloracetyl moiety is an aliphatic haloacetyl side-chain and the propane­diol moiety possesses two stereogenic carbon atoms (C7 and C8), carrying the hydroxyl group and the amide side chain, respectively. In the arbitrarily chosen asymmetric mol­ecule of (**I**), C7 and C8 both have *R* configuration, but crystal symmetry generates a racemic mixture. In natural, chiral, chloramphenicol, the equivalent atoms also have *R* configurations. In the *p*-nitro­benzene moiety in (**I**), the nitro (N1/O1/O2) group is oriented at a dihedral angle of 4.3 (3)° with respect the the C1–C6 benzene ring and atoms N1 and C7 are −0.037 (3) and −0.041 (2) Å away from the benzene ring plane. In the dichloracetyl moiety, atoms Cl3 and Cl4 are −1.3541 (8) and 1.5347 (8) Å, respectively, away from the best plane of the O5/N2/C10/C11 acetyl group (r.m.s. deviation = 0.008 Å). In the 2-amino-propane­diol moiety, the dihedral angles between the *A* (O4/N2/C8/C9) [r.m.s. deviation = 0.013Å], *B* (N2/C7/C8) and *C* (O3/C7/C8) fragments are *A*/*B* = 56.46 (16)°, *A*/*C* = 77.92 (9)° and *B*/*C* = 43.64 (18)°. On the other hand, the O3—C7—C8—C9, O3—C7—C8—N2 and O4—C9—C8—N2 torsion angles are −78.3 (2), 43.6 (2) and 177.91 (16)°, respectively. The bond lengths and angles in the title triclinic polymorph are significantly different from the corresponding values in the ortho­rhom­bic polymorph of chloramphenicol (Acharya *et al.*, 1979 ▸; Chatterjee *et al.*, 1979 ▸; Dunitz, 1952 ▸)

## 3. Supra­molecular features

**Figure 2:**
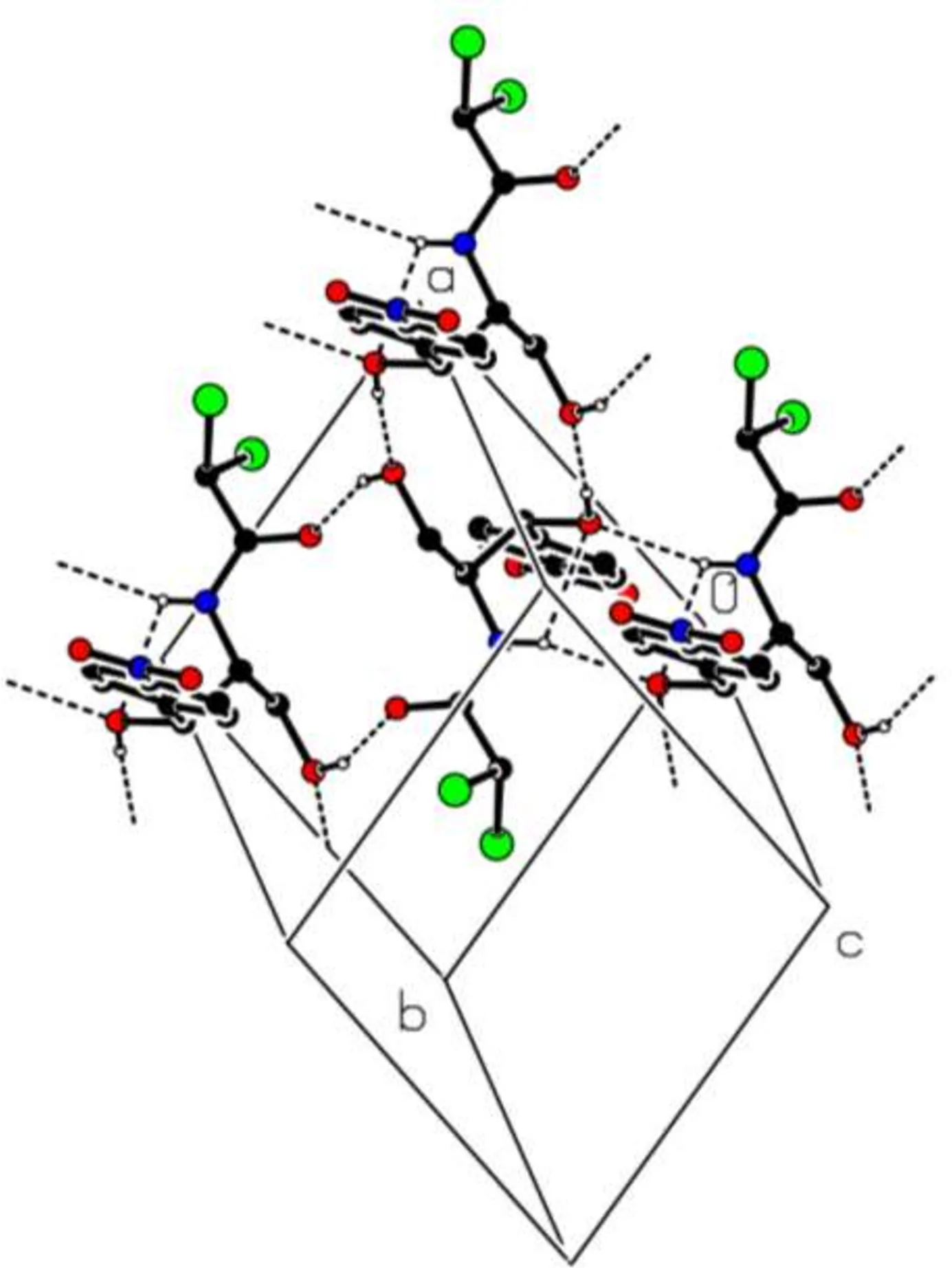
A partial packing diagram of (**I**) showing the inter­molecular N—H⋯O and O—H⋯O hydrogen bonds as dashed lines.

In the extended structure of (**I**), O—H⋯O and N—H⋯O hydrogen bonds (Table 1 ▸) link the mol­ecules, enclosing 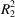
(12), 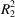
(14) and 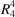
(4) ring motifs, into a three-dimensional network (Figs. 2 ▸ and 3 ▸). The packing is consolidated by C—H⋯O hydrogen bonds and aromatic π–π stacking inter­actions between the benzene rings with centroid- to-centroid distance of 3.7524 (13) Å (slippage = 1.330 Å). Unlike the intra­molecular O—H⋯O hydrogen bond in natural chloramphenicol (Staples, 2022 ▸; CSD refcode CLMPCL04), in (**I**), both –OH groups participate in inter­molecular O—H⋯O inter­actions with O⋯O contact distances of 2.706 (2) Å and 2.780 (2) Å (Table 1 ▸).

**Figure 4:**
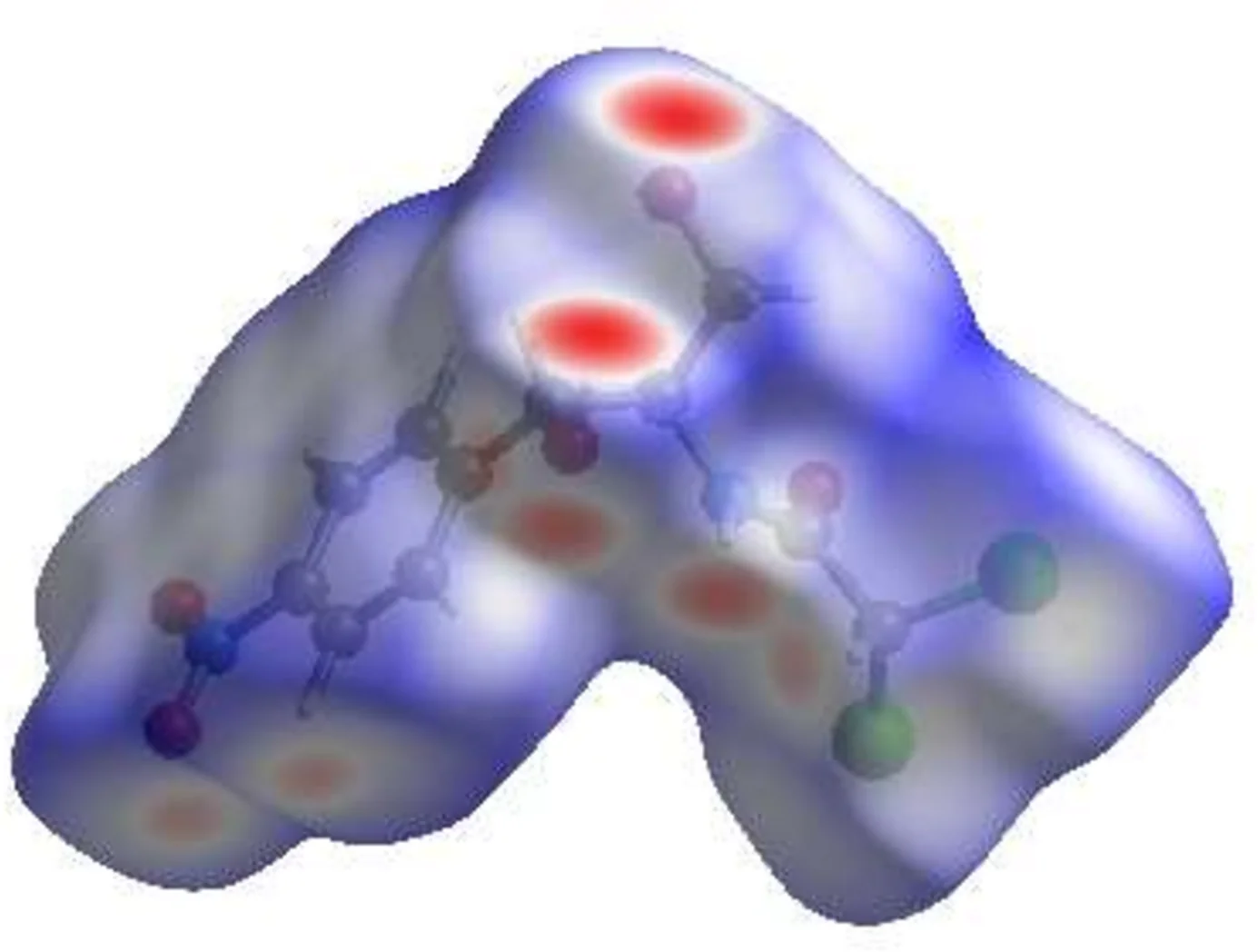
View of the three-dimensional Hirshfeld surface for (**I**) plotted over *d*_norm_ in the range from −0.67 to 1.50 a.u.

**Figure 5:**
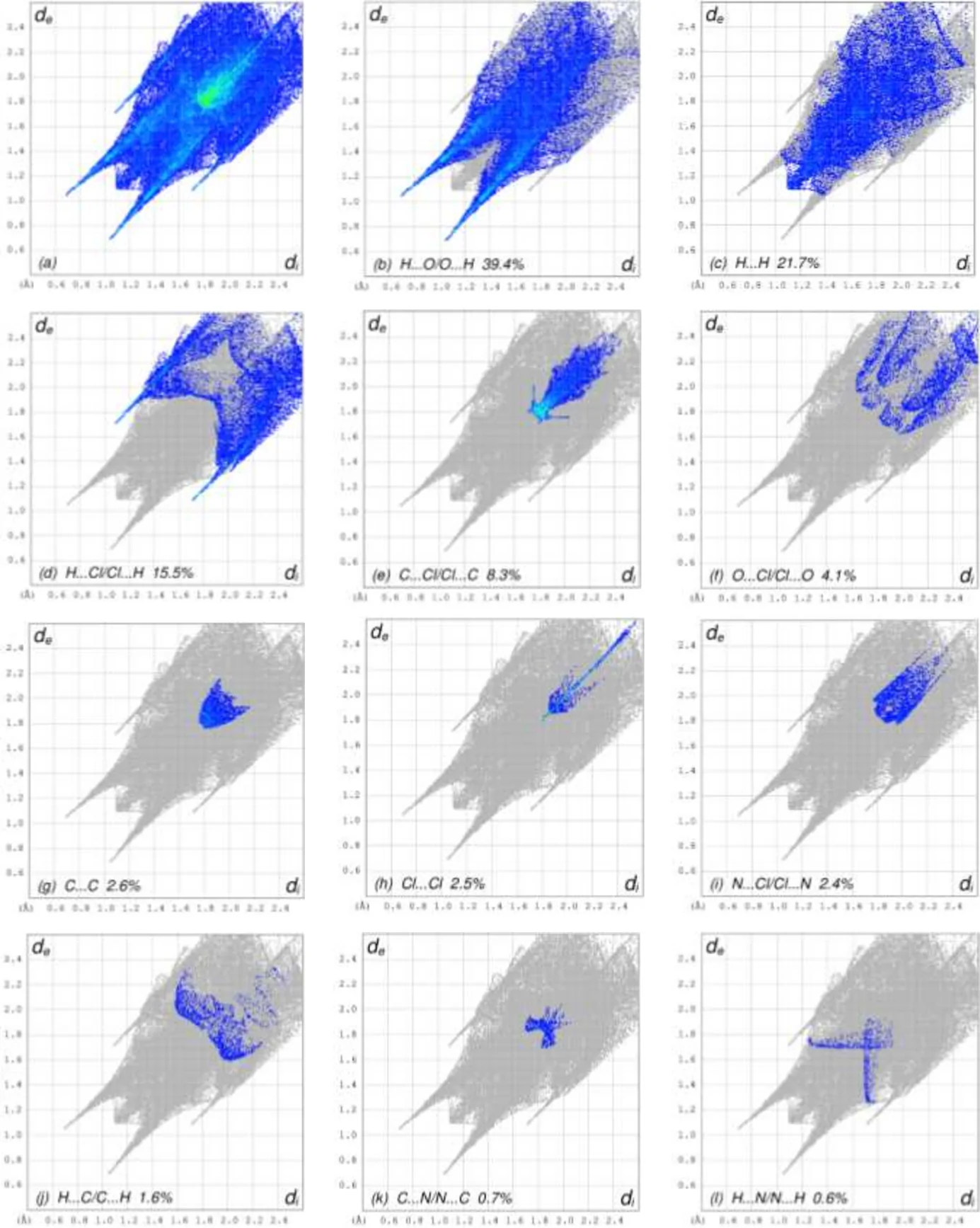
The two-dimensional fingerprint plots for (**I**), showing (*a*) all inter­actions, and delineated into different contact types (*b*)–(n). The *d*_i_ and *d*_e_ values are the closest inter­nal and external distances (in Å) from given points on the Hirshfeld surface.

The inter­molecular inter­actions in the crystal of (**I**) were visualized by carrying out a Hirshfeld surface (HS) analysis using *CrystalExplorer* 17.5 (Spackman *et al.*, 2021 ▸). The red spots (Fig. 4 ▸) indicate their roles as the respective donors and/or acceptors atoms in hydrogen bonding, as discussed above. The overall two-dimensional fingerprint plot is shown in Fig. 5 ▸*a* and those delineated into different contact types in Fig. 5 ▸*b*–*n*. According to these data, the H⋯O/O⋯H, H⋯H, H⋯C/C⋯H and C⋯Cl/Cl⋯Cl contacts make the most significant contributions to the HS, at 39.4%, 21.7%, 15.5% and 8.3%, respectively (Fig. 7).

The volume of the crystal voids (see figure in the supporting information) and the percentage of free space in the unit cell of (**I**) are 115.2 Å^3^ and 16.3%, respectively. These values compare with 324.1 Å^3^ and 11.9%, respectively in CLMPCL04, which suggests that the homochiral mol­ecules in CLMPCL04 pack more effectively in space group *C*222_1_ than do the racemic mol­ecules in space group *P*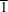 in (**I**). This is supported by the difference in unit-cell volumes [707.30 (3) Å^3^ for (**I**) (*Z* = 2) and 2733.30 (6) Å^3^ for CLMPCL04 (*Z* = 8)], although it should be noted that the intensity data for (**I**) were collected at 292 K and those for CLMPCL04 at 100 K.

For further computational chemistry results (electrostatic potential, shape-index, void volume figures, inter­action energies), see the supporting information.

## 4. Database survey

A survey of the Cambridge Structural Database (CSD, July 2025 update; Groom *et al.*, 2016 ▸) revealed six structures of natural chloramphenicol: CSD refcode CLMPCL (Sundaralingam *et al.*, 1971 ▸), CLMPCL01 (Acharya *et al.*, 1979 ▸), CLMPCL02 (Chatterjee *et al.*, 1979 ▸), CLMPCL03 (Dunitz, 1952 ▸), CLMPCL04 (Staples, 2022 ▸) and EJILUH (Ma *et al.*, 2020 ▸). The first five crystallize in space group *C*222_1_ with *a* ≃ 7.34, *b* ≃ 17.34, *c* ≃ 21.49 Å (or equivalent setting of the unit cell) and the final structure is a clathrate.

## 5. Synthesis and crystallization

A commercial sample of chloramphenicol was recrystallised by slow evaporation of an ethanol–water (1:1 *v*/*v*) solution at room temperature.

## 6. Refinement

Crystal data, data collection and structure refinement details are summarized in Table 2 ▸. The NH hydrogen atom was located from a difference Fourier map and refined isotropically. The O- and C-bound hydrogen-atom positions were calculated geometrically at distances of 0.82 Å (for OH), 0.93–0.98 Å (for CH), and refined using a riding model with the constraint of U_iso_ = *k* × U_eq_ (C, O), where *k* = 1.5 for OH hydrogen atoms and *k* = 1.2 for other hydrogen atoms.

## Supplementary Material

Crystal structure: contains datablock(s) I, global. DOI: 10.1107/S2056989026008765/hb8237sup1.cif

Structure factors: contains datablock(s) I. DOI: 10.1107/S2056989026008765/hb8237Isup2.hkl

Electrostatic potential. DOI: 10.1107/S2056989026008765/hb8237sup3.docx

Shape index. DOI: 10.1107/S2056989026008765/hb8237sup4.docx

Void volume. DOI: 10.1107/S2056989026008765/hb8237sup5.docx

Energy framework. DOI: 10.1107/S2056989026008765/hb8237sup6.docx

Supporting information file. DOI: 10.1107/S2056989026008765/hb8237Isup7.cml

CCDC reference: 2582935

Additional supporting information:  crystallographic information; 3D view; checkCIF report

## Figures and Tables

**Figure 3:**
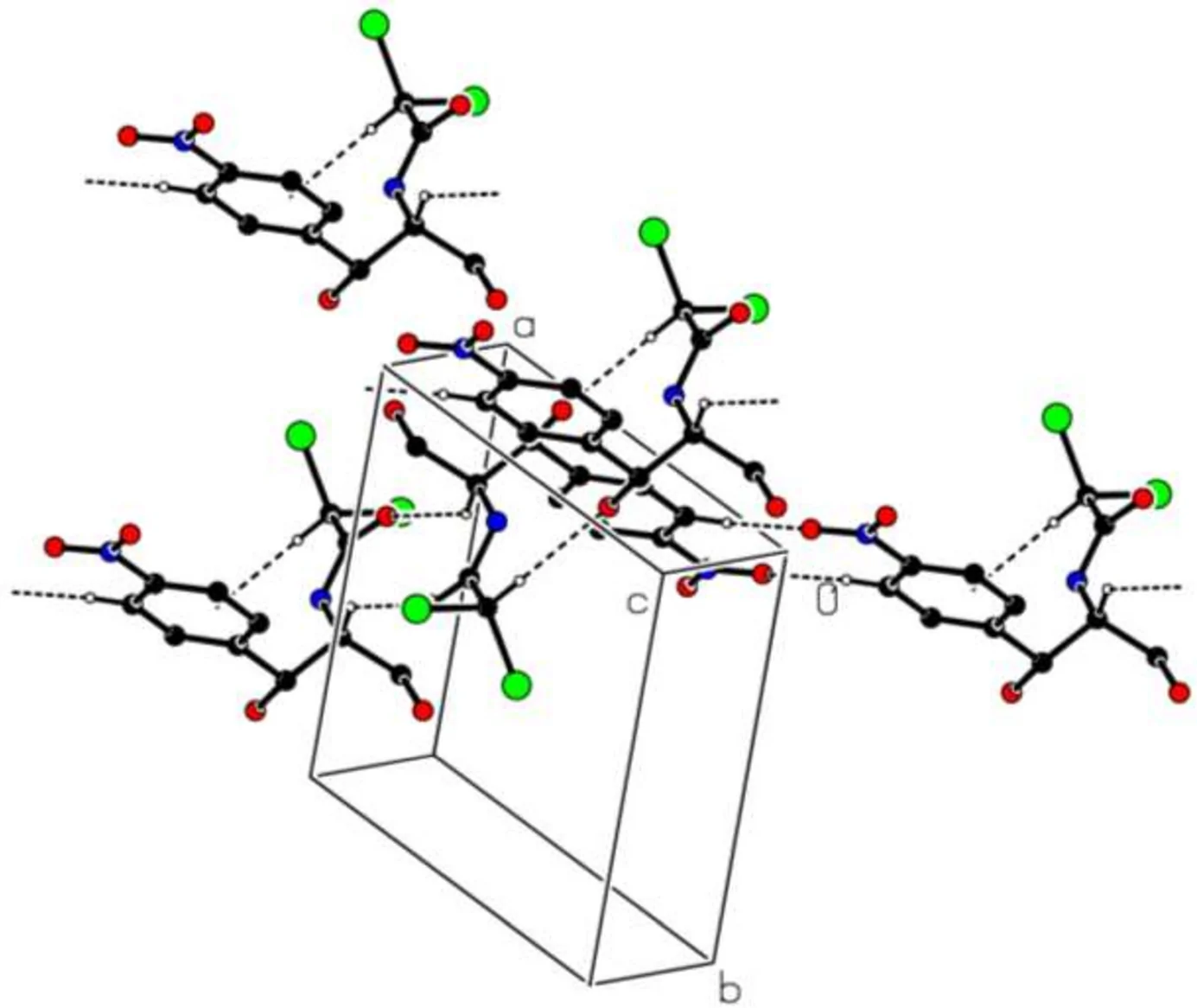
A partial packing diagram of (**I**) showing C—H⋯O hydrogen bonds as dashed lines.

**Table 1:** Hydrogen-bond geometry (Å, °)

*D*—H⋯*A*	*D*—H	H⋯*A*	*D*⋯*A*	*D*—H⋯*A*
O4—H4⋯O5^i^	0.82	2.00	2.780 (2)	159
O3—H3⋯O4^ii^	0.82	1.90	2.706 (2)	170
N2—H2⋯O3^iii^	0.83 (3)	2.23 (3)	3.010 (2)	155 (2)
C11—H11⋯O3^iii^	0.98	2.42	3.302 (3)	149
C8—H8⋯O5^i^	0.98	2.39	3.163 (2)	135
C3—H3*A*⋯O1^iv^	0.93	2.46	3.298 (4)	151

Symmetry codes: (i) 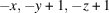; (ii) 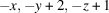; (iii) 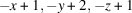; (iv) 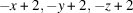.

**Table 2:** Experimental details

Crystal data	
Chemical formula	C_11_H_12_Cl_2_N_2_O_5_
*M*_r_	323.13
Crystal system, space group	Triclinic, *P* 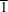
Temperature (K)	292
*a*, *b*, *c* (Å)	8.0656 (2), 8.9248 (2), 11.0003 (2)
α, β, γ (°)	97.522 (2), 105.938 (2), 107.280 (2)
*V* (Å^3^)	707.30 (3)
*Z*	2
Radiation type	Cu *K*α
μ (mm^−1^)	4.34
Crystal size (mm)	0.3 × 0.24 × 0.15

Data collection	
Diffractometer	XtaLAB Synergy, Single source at home/near, HyPix3000
Absorption correction	Multi-scan ((*CrysAlis PRO*; Rigaku OD, 2023 ▸)
*T*_min_, *T*_max_	0.656, 1.000
No. of measured, independent and observed [*I* > 2σ(*I*)] reflections	5967, 2718, 2507
*R*_int_	0.034
(sin θ/λ)_max_ (Å^−1^)	0.615

Refinement	
*R*[*F*^2^ > 2σ(*F*^2^)], *wR*(*F*^2^), *S*	0.052, 0.141, 1.05
No. of reflections	2718
No. of parameters	188
H-atom treatment	H atoms treated by a mixture of independent and constrained refinement
Δρ_max_, Δρ_min_ (e Å^−3^)	0.59, −0.68

Computer programs: *CrysAlis PRO* (Rigaku OD, 2023 ▸), *SHELXT2014/5* (Sheldrick, 2015*a* ▸), *SHELXL2016/6* (Sheldrick, 2015*b* ▸) and *OLEX2*a (Dolomanov *et al.*, 2009 ▸).

## supplementary crystallographic information

### 2,2-Dichloro-N-[(1R,2R)-1,3-dihydroxy-1-(4-nitrophenyl)propan-2-yl]acetamide Crystal data

**Table:**

C_11_H_12_Cl_2_N_2_O_5_	*Z* = 2
*M**_r_* = 323.13	*F*(000) = 332
Triclinic, *P*1	*D*_x_ = 1.517 Mg m^−^^3^
*a* = 8.0656 (2) Å	Cu *K*α radiation, λ = 1.54184 Å
*b* = 8.9248 (2) Å	Cell parameters from 4694 reflections
*c* = 11.0003 (2) Å	θ = 4.3–71.5°
α = 97.522 (2)°	µ = 4.34 mm^−^^1^
β = 105.938 (2)°	*T* = 292 K
γ = 107.280 (2)°	Block, colourless
*V* = 707.30 (3) Å^3^	0.3 × 0.24 × 0.15 mm

### 2,2-Dichloro-N-[(1R,2R)-1,3-dihydroxy-1-(4-nitrophenyl)propan-2-yl]acetamide Data collection

**Table:**

XtaLAB Synergy, Single source at home/near, HyPix3000 diffractometer	2718 independent reflections
Radiation source: micro-focus sealed X-ray tube, PhotonJet (Cu) X-ray Source	2507 reflections with *I* > 2σ(*I*)
Mirror monochromator	*R*_int_ = 0.034
Detector resolution: 10.0000 pixels mm^-1^	θ_max_ = 71.4°, θ_min_ = 4.3°
ω scans	*h* = −9→8
Absorption correction: multi-scan ((CrysAlisPro; Rigaku OD, 2023)	*k* = −10→10
*T*_min_ = 0.656, *T*_max_ = 1.000	*l* = −10→13
5967 measured reflections	

### 2,2-Dichloro-N-[(1R,2R)-1,3-dihydroxy-1-(4-nitrophenyl)propan-2-yl]acetamide Refinement

**Table:**

Refinement on *F*^2^	Hydrogen site location: mixed
Least-squares matrix: full	H atoms treated by a mixture of independent and constrained refinement
*R*[*F*^2^ > 2σ(*F*^2^)] = 0.052	*w* = 1/[σ^2^(*F*_o_^2^) + (0.0747*P*)^2^ + 0.5048*P*] where *P* = (*F*_o_^2^ + 2*F*_c_^2^)/3
*wR*(*F*^2^) = 0.141	(Δ/σ)_max_ < 0.001
*S* = 1.05	Δρ_max_ = 0.59 e Å^−^^3^
2718 reflections	Δρ_min_ = −0.68 e Å^−^^3^
188 parameters	Extinction correction: SHELXL-2016/6 (Sheldrick 2016), Fc^*^=kFc[1+0.001xFc^2^λ^3^/sin(2θ)]^-1/4^
0 restraints	Extinction coefficient: 0.0140 (14)
Primary atom site location: dual	

### 2,2-Dichloro-N-[(1R,2R)-1,3-dihydroxy-1-(4-nitrophenyl)propan-2-yl]acetamide Special details

**Table:**

Geometry. All esds (except the esd in the dihedral angle between two l.s. planes) are estimated using the full covariance matrix. The cell esds are taken into account individually in the estimation of esds in distances, angles and torsion angles; correlations between esds in cell parameters are only used when they are defined by crystal symmetry. An approximate (isotropic) treatment of cell esds is used for estimating esds involving l.s. planes.

### 2,2-Dichloro-N-[(1R,2R)-1,3-dihydroxy-1-(4-nitrophenyl)propan-2-yl]acetamide Fractional atomic coordinates and isotropic or equivalent isotropic displacement parameters (Å^2^)

**Table:**

	*x*	*y*	*z*	*U*_iso_*/*U*_eq_	
Cl3	0.47682 (11)	0.44633 (10)	0.32614 (7)	0.0589 (3)	
Cl4	0.19337 (11)	0.49969 (11)	0.12769 (7)	0.0643 (3)	
O4	−0.1306 (2)	0.79888 (19)	0.50133 (18)	0.0361 (4)	
H4	−0.151263	0.726781	0.540228	0.054*	
O3	0.3697 (2)	1.04729 (17)	0.57744 (15)	0.0286 (4)	
H3	0.302019	1.094821	0.545594	0.043*	
O5	0.1354 (2)	0.46694 (18)	0.39138 (18)	0.0389 (4)	
O1	0.8908 (3)	0.8840 (4)	1.1205 (2)	0.0880 (10)	
O2	0.6514 (4)	0.7306 (4)	1.1442 (2)	0.0762 (8)	
N1	0.7258 (3)	0.8144 (3)	1.0840 (2)	0.0472 (6)	
N2	0.2773 (2)	0.7375 (2)	0.44290 (17)	0.0247 (4)	
H2	0.355 (4)	0.814 (3)	0.430 (2)	0.026 (6)*	
C11	0.3536 (3)	0.5760 (3)	0.2873 (2)	0.0335 (5)	
H11	0.439319	0.683345	0.294084	0.040*	
C10	0.2461 (3)	0.5879 (2)	0.3806 (2)	0.0272 (4)	
C8	0.1641 (3)	0.7676 (2)	0.51967 (19)	0.0230 (4)	
H8	0.131957	0.677095	0.560800	0.028*	
C2	0.5884 (3)	0.9648 (3)	0.7853 (2)	0.0345 (5)	
H2A	0.643424	1.030383	0.737875	0.041*	
C7	0.2722 (3)	0.9228 (2)	0.6273 (2)	0.0243 (4)	
H7	0.182310	0.958821	0.655455	0.029*	
C4	0.6133 (3)	0.8411 (3)	0.9648 (2)	0.0349 (5)	
C9	−0.0134 (3)	0.7752 (3)	0.4297 (2)	0.0305 (5)	
H9A	0.015934	0.863249	0.386989	0.037*	
H9B	−0.076981	0.675638	0.363211	0.037*	
C5	0.4241 (3)	0.7678 (3)	0.9267 (2)	0.0377 (5)	
H5	0.369825	0.701347	0.973919	0.045*	
C6	0.3185 (3)	0.7963 (3)	0.8166 (2)	0.0348 (5)	
H6	0.191082	0.748704	0.789635	0.042*	
C1	0.3986 (3)	0.8948 (2)	0.7449 (2)	0.0267 (4)	
C3	0.6970 (3)	0.9380 (3)	0.8960 (2)	0.0399 (6)	
H3A	0.824555	0.984839	0.923189	0.048*	

### 2,2-Dichloro-N-[(1R,2R)-1,3-dihydroxy-1-(4-nitrophenyl)propan-2-yl]acetamide Atomic displacement parameters (Å^2^)

**Table:**

	*U*^11^	*U*^22^	*U*^33^	*U*^12^	*U*^13^	*U*^23^
Cl3	0.0632 (5)	0.0683 (5)	0.0572 (5)	0.0432 (4)	0.0213 (4)	0.0035 (3)
Cl4	0.0564 (5)	0.0918 (6)	0.0335 (4)	0.0162 (4)	0.0097 (3)	0.0113 (3)
O4	0.0248 (8)	0.0325 (8)	0.0591 (11)	0.0137 (6)	0.0178 (7)	0.0201 (7)
O3	0.0227 (7)	0.0236 (7)	0.0407 (8)	0.0074 (6)	0.0111 (6)	0.0115 (6)
O5	0.0389 (9)	0.0249 (8)	0.0556 (11)	0.0050 (7)	0.0257 (8)	0.0110 (7)
O1	0.0376 (12)	0.147 (3)	0.0639 (15)	0.0123 (14)	−0.0005 (11)	0.0559 (16)
O2	0.0597 (14)	0.118 (2)	0.0545 (13)	0.0253 (14)	0.0152 (11)	0.0530 (14)
N1	0.0421 (13)	0.0662 (15)	0.0320 (11)	0.0186 (11)	0.0086 (9)	0.0157 (10)
N2	0.0229 (9)	0.0217 (8)	0.0311 (9)	0.0050 (7)	0.0134 (7)	0.0079 (7)
C11	0.0325 (12)	0.0288 (11)	0.0363 (12)	0.0062 (9)	0.0148 (9)	0.0006 (9)
C10	0.0243 (10)	0.0244 (10)	0.0328 (11)	0.0071 (8)	0.0099 (8)	0.0079 (8)
C8	0.0210 (10)	0.0214 (9)	0.0280 (10)	0.0066 (7)	0.0102 (8)	0.0071 (7)
C2	0.0266 (11)	0.0386 (12)	0.0354 (12)	0.0058 (9)	0.0099 (9)	0.0125 (9)
C7	0.0201 (9)	0.0242 (9)	0.0302 (10)	0.0074 (7)	0.0115 (8)	0.0061 (8)
C4	0.0344 (12)	0.0445 (13)	0.0243 (11)	0.0151 (10)	0.0069 (9)	0.0066 (9)
C9	0.0220 (10)	0.0312 (11)	0.0354 (11)	0.0076 (8)	0.0064 (9)	0.0092 (9)
C5	0.0380 (13)	0.0455 (13)	0.0308 (11)	0.0103 (10)	0.0160 (10)	0.0134 (10)
C6	0.0261 (11)	0.0439 (13)	0.0339 (12)	0.0086 (9)	0.0123 (9)	0.0105 (10)
C1	0.0267 (11)	0.0266 (10)	0.0265 (10)	0.0095 (8)	0.0098 (8)	0.0023 (8)
C3	0.0257 (11)	0.0493 (14)	0.0378 (12)	0.0079 (10)	0.0052 (9)	0.0107 (10)

### 2,2-Dichloro-N-[(1R,2R)-1,3-dihydroxy-1-(4-nitrophenyl)propan-2-yl]acetamide Geometric parameters (Å, º)

**Table:**

Cl3—C11	1.758 (2)	C8—C7	1.538 (3)
Cl4—C11	1.771 (2)	C8—C9	1.527 (3)
O4—H4	0.8200	C2—H2A	0.9300
O4—C9	1.429 (3)	C2—C1	1.384 (3)
O3—H3	0.8200	C2—C3	1.387 (3)
O3—C7	1.425 (2)	C7—H7	0.9800
O5—C10	1.223 (3)	C7—C1	1.512 (3)
O1—N1	1.212 (3)	C4—C5	1.384 (3)
O2—N1	1.199 (3)	C4—C3	1.371 (4)
N1—C4	1.469 (3)	C9—H9A	0.9700
N2—H2	0.83 (3)	C9—H9B	0.9700
N2—C10	1.334 (3)	C5—H5	0.9300
N2—C8	1.459 (3)	C5—C6	1.378 (3)
C11—H11	0.9800	C6—H6	0.9300
C11—C10	1.527 (3)	C6—C1	1.391 (3)
C8—H8	0.9800	C3—H3A	0.9300
			
C9—O4—H4	109.5	O3—C7—H7	107.4
C7—O3—H3	109.5	O3—C7—C1	111.64 (16)
O1—N1—C4	118.1 (2)	C8—C7—H7	107.4
O2—N1—O1	122.5 (2)	C1—C7—C8	112.27 (16)
O2—N1—C4	119.3 (2)	C1—C7—H7	107.4
C10—N2—H2	118.5 (17)	C5—C4—N1	118.1 (2)
C10—N2—C8	120.61 (17)	C3—C4—N1	119.7 (2)
C8—N2—H2	120.5 (17)	C3—C4—C5	122.2 (2)
Cl3—C11—Cl4	110.43 (12)	O4—C9—C8	110.65 (18)
Cl3—C11—H11	109.6	O4—C9—H9A	109.5
Cl4—C11—H11	109.6	O4—C9—H9B	109.5
C10—C11—Cl3	109.68 (16)	C8—C9—H9A	109.5
C10—C11—Cl4	107.81 (16)	C8—C9—H9B	109.5
C10—C11—H11	109.6	H9A—C9—H9B	108.1
O5—C10—N2	124.1 (2)	C4—C5—H5	121.0
O5—C10—C11	120.72 (19)	C6—C5—C4	118.0 (2)
N2—C10—C11	115.15 (18)	C6—C5—H5	121.0
N2—C8—H8	108.4	C5—C6—H6	119.3
N2—C8—C7	110.40 (16)	C5—C6—C1	121.4 (2)
N2—C8—C9	109.22 (17)	C1—C6—H6	119.3
C7—C8—H8	108.4	C2—C1—C7	123.15 (19)
C9—C8—H8	108.4	C2—C1—C6	119.0 (2)
C9—C8—C7	112.08 (16)	C6—C1—C7	117.86 (19)
C1—C2—H2A	119.7	C2—C3—H3A	120.6
C1—C2—C3	120.5 (2)	C4—C3—C2	118.9 (2)
C3—C2—H2A	119.7	C4—C3—H3A	120.6
O3—C7—C8	110.49 (16)		
			
Cl3—C11—C10—O5	−57.1 (3)	C10—N2—C8—C9	−82.3 (2)
Cl3—C11—C10—N2	125.53 (18)	C8—N2—C10—O5	−6.5 (3)
Cl4—C11—C10—O5	63.2 (3)	C8—N2—C10—C11	170.81 (18)
Cl4—C11—C10—N2	−114.18 (18)	C8—C7—C1—C2	115.6 (2)
O3—C7—C1—C2	−9.1 (3)	C8—C7—C1—C6	−65.8 (2)
O3—C7—C1—C6	169.51 (18)	C7—C8—C9—O4	−59.4 (2)
O1—N1—C4—C5	−176.9 (3)	C4—C5—C6—C1	0.4 (4)
O1—N1—C4—C3	2.4 (4)	C9—C8—C7—O3	−78.3 (2)
O2—N1—C4—C5	0.1 (4)	C9—C8—C7—C1	156.31 (17)
O2—N1—C4—C3	179.4 (3)	C5—C4—C3—C2	0.7 (4)
N1—C4—C5—C6	178.4 (2)	C5—C6—C1—C2	0.2 (4)
N1—C4—C3—C2	−178.5 (2)	C5—C6—C1—C7	−178.5 (2)
N2—C8—C7—O3	43.6 (2)	C1—C2—C3—C4	−0.1 (4)
N2—C8—C7—C1	−81.7 (2)	C3—C2—C1—C7	178.3 (2)
N2—C8—C9—O4	177.91 (16)	C3—C2—C1—C6	−0.4 (4)
C10—N2—C8—C7	154.03 (18)	C3—C4—C5—C6	−0.9 (4)

### 2,2-Dichloro-N-[(1R,2R)-1,3-dihydroxy-1-(4-nitrophenyl)propan-2-yl]acetamide Hydrogen-bond geometry (Å, º)

**Table:**

*D*—H···*A*	*D*—H	H···*A*	*D*···*A*	*D*—H···*A*
O4—H4···O5^i^	0.82	2.00	2.780 (2)	159
O3—H3···O4^ii^	0.82	1.90	2.706 (2)	170
N2—H2···O3^iii^	0.83 (3)	2.23 (3)	3.010 (2)	155 (2)
C11—H11···O3^iii^	0.98	2.42	3.302 (3)	149
C8—H8···O5^i^	0.98	2.39	3.163 (2)	135
C3—H3*A*···O1^iv^	0.93	2.46	3.298 (4)	151

Symmetry codes: (i) −*x*, −*y*+1, −*z*+1; (ii) −*x*, −*y*+2, −*z*+1; (iii) −*x*+1, −*y*+2, −*z*+1; (iv) −*x*+2, −*y*+2, −*z*+2.
