# Supplementary material for: Crystal structure of racemic chloramphenicol
Source: Acta Crystallogr E Crystallogr Commun. 2026 Aug 28;82(Pt 9):1091–4. doi: 10.1107/S2056989026008765 (PMC13548768; doi:10.1107/S2056989026008765)
Supplement: Supplementary file 1 [file e-82-01091-sup3.docx]

**Figure 5**

View of the three-dimensional Hirshfeld surface of the title compound plotted over electrostatic potential in the range of −0.0500 to 0.0500 a.u. using the STO-3 G basis set at the Hartree-Fock level of theory. Hydrogen-bond donors and acceptors are shown as blue and red regions around the atoms, corresponding to positive and negative potentials, respectively.
