# Supplementary material for: Crystal structure of racemic chloramphenicol
Source: Acta Crystallogr E Crystallogr Commun. 2026 Aug 28;82(Pt 9):1091–4. doi: 10.1107/S2056989026008765 (PMC13548768; doi:10.1107/S2056989026008765)
Supplement: Supplementary file 4 [file e-82-01091-sup6.docx]

(a)	(b)	(c)

**Figure 9**

The energy frameworks for a cluster of molecules of the title compound viewed down the *a*-axis showing the (*a*) electrostatic energy, (*b*) dispersion energy and (*c*) total energy diagrams. The cylindirical radius is proportional to the relative strength of the corresponding energies and they were adjusted to the same scale factor of 80 with cut-off value of 5 kJ mol^−1^ within 2 *X* 2 *X* 2 unit cells.
