# Supplementary material for: Crystal structure of racemic chloramphenicol
Source: Acta Crystallogr E Crystallogr Commun. 2026 Aug 28;82(Pt 9):1091–4. doi: 10.1107/S2056989026008765 (PMC13548768; doi:10.1107/S2056989026008765)
Supplement: Supplementary file 5 [file e-82-01091-sup4.docx]

**Figure 6**

The shape index showing the π···π interactions.
