# Supplementary material for: Crystal structure of racemic chloramphenicol
Source: Acta Crystallogr E Crystallogr Commun. 2026 Aug 28;82(Pt 9):1091–4. doi: 10.1107/S2056989026008765 (PMC13548768; doi:10.1107/S2056989026008765)
Supplement: Supplementary file 6 [file e-82-01091-sup5.docx]

(a)	(b)	(c)

**Figure 8**

Crystal voids viewed down the (*a*) *a*-axis, (*b*) *b*- axis and (*c*) *c*-axis directions.
